# Genetic Diversity and Differentiation of *Juniperus thurifera* in Spain and Morocco as Determined by SSR

**DOI:** 10.1371/journal.pone.0088996

**Published:** 2014-02-12

**Authors:** Helena Teixeira, Susana Rodríguez-Echeverría, Cristina Nabais

**Affiliations:** Centro de Ecologia Funcional, Departamento de Ciências da Vida, Universidade de Coimbra, Coimbra, Portugal; University of Milano Bicocca, Italy

## Abstract

*Juniperus thurifera* L. is an important tree endemic to the western Mediterranean basin that it is able to grow in semi-arid climates. It nowadays exhibits a disjunct distribution pattern, occurring in North Africa, Spain, France and the Italian Alps. The Strait of Gibraltar has acted as an efficient barrier against gene flow between African and European populations, which are considered different subspecies by some authors. We aimed at describing the intraspecific genetic diversity of *J. thurifera* in populations from the Iberian Peninsula and Morocco and the phylogeographical relationships among these populations. The ploidy level of *J. thurifera* was examined and eleven nuclear microsatellites (nSSRs) developed for *J. thurifera* were assessed for genotyping this species. Six nSSRs were polymorphic and subsequently used to assess the genetic diversity and structure of the studied populations. Genotyping of the tetraploid *J. thurifera* using nuclear microsatellites supports the separation of Moroccan and Spanish populations into two genetically differentiated groups that correspond to the proposed subspecies *africana* and *thurifera*. High values of within population genetic diversity were found, that accounted for 90% of the total genetic variance, while population structure was weak. The estimators of genetic diversity were higher in populations of Spain than in populations of Morocco pointing for a possible loss of genetic diversity during the spread of this species to Africa from Europe.

## Introduction

*Juniperus thurifera* L. (section *Sabina*) is a dioecious tree endemic to the western Mediterranean basin [1]. It is a long-lived species with individual trees lasting for several hundred years [2]. *Juniperus thurifera* was likely widespread during cold stages of the Pleistocene but nowadays it exhibits a disjunct distribution pattern, occurring in North Africa (Morocco and Algeria), Spain, France (Pyrenees, Alps and Corsica) and in the Italian Alps [3], [4]. The most abundant populations are in Spain (>200,000 ha) and the Medium and High Atlas Mountains in Morocco (30,000 ha). Fragmentation of *J. thurifera* populations was initially driven by increased aridity and warmer climates, but it is currently caused by human activities such as intense wood removal and grazing in North Africa and land use changes in the Iberian Peninsula that lead to the colonization of *Juniperus* stands by pines or oaks [3], [5]. *Juniperus thurifera* is most often found in calcareous soils in the Iberian Peninsula and acidic soils in North Africa [3]. It is an important component of woodland communities of cold and dry sites from 200 to 1800 m above sea level in the Iberian Peninsula, and in sites with subhumid cold winter climates mainly between 1800 and 3150 m above sea level in Morocco [3], [6].

*Juniperus thurifera* is a morphologically variable species due to the long-term isolation of disjunct populations. The Strait of Gibraltar has acted as an efficient barrier against gene flow between Morocco and European populations for a long time [4]. Consequently, two subspecies have been described: subsp. *thurifera* in Europe and subsp. *africana* in Africa. *Juniperus thurifera* subsp. *africana* (Maire) Romo et Boratyński differs from *J. thurifera* subsp. *thurifera* by its smaller cone diameter (5–6 vs. 7–12 mm), lower number of seeds per cone (only 1–2 vs. 2–5) and shorter leaf scales (1–1.5 vs. 2–2.5 mm) [3], [7]. Studies based on essential oils [8] and biochemical composition [9] supported the clear differentiation of populations from Europe and populations from Morocco. The existence of the two subspecies has been confirmed also by phylogeographic studies based on RAPD [10] and AFLP markers [4]. However, not all authors agree upon the distinctiveness of subsp. *africana*. Farjon [11] considers that the biochemical composition is taxonomically uninformative and cannot be used to justify this distinction, and a clear separation of morphological traits is not always clear. Maire [12] cited specimens from Algeria with bigger cone diameter (9 mm) and material from Spain with short leaves similar to the African populations. Also, populations from Algeria do not cluster with African populations but are genetically intermediate between Spanish and Moroccan populations [4].

A number of PCR-based techniques can be used to detect polymorphisms for studies of genetic diversity, being AFLP (Amplified Fragment Length Polymorphism) and SSR (Simple Sequence Repeat or microsatellites) the most popular molecular markers used [13]. Some authors have compared the data produced by AFLP and SSR and showed that both markers have comparable efficiency [14], [15]. However, SSR could be more informative because they are co-dominant markers (unlike AFLPs that are dominant markers), and the heterozygosity of polymorphic loci for SSR is greater than for AFLP because replication slippage occurs more frequently than single nucleotide mutations and insertion/deletion events [15]. In addition, using SSRs makes possible the analysis of problematic samples, like dry leaves, because the targeted DNA fragments have a smaller size than those in AFLP.

In the present study, we used nuclear microsatellites (nSSRs) to assess the genetic structure of *J. thurifera* populations from the Iberian Peninsula and Morocco. Specifically, our aims were (1) to analyse the ploidy level of *J. thurifera* in both sides of the Strait of Gibraltar, (2) to describe the intraspecific genetic diversity of *J. thurifera* in populations from the Iberian Peninsula and Morocco, and (3) to study the phylogeographical relationships among these populations.

## Materials and Methods

### Plant material

Fresh leaves were collected from 20–30 individuals selected randomly in 11 natural populations of *Juniperus thurifera*. Five populations were sampled in Morocco and six in Spain. A total of 261 individuals were included in the study. Table S1 gives the details of the geographical location of populations and number of sampled individuals per population. Special permissions for sampling were not required since this is not a protected species, sampling was conducted outside protected areas and leaf collection does not represent a threat for the sampled individuals.

### DNA extraction

The leaves collected from each individual were dried at 30°C overnight before DNA extraction. Genomic DNA was extracted from 20 mg of leaves using the DNeasy Plant Mini Kit (Qiagen). DNA extraction followed the manufacturer's protocol with a few modifications: instead of 200 µl of buffer AE we only added 150 µl and the time of incubation was extended to 10–15 min. The quality of the extracted DNA was checked on 1% TBE agarose gels stained with RedSafe. DNA concentration was estimated using NanoDrop 2000c (Thermo Scientific, Wilmington, USA) and samples were stored at −20°C.

### Flow cytometry analysis

The genome size of *J. thurifera* was inferred using flow cytometry following Castro et al. [16]. Nuclear suspensions were obtained by chopping leaf tissue of *J. thurifera*, *J. phoenicea* and *Vicia faba* with a razor blade in a Petri dish containing 1 ml of WPB buffer (0.2 M Tris–HCl, 4 mM MgCl2·6H2O, 1% Triton X-100, 2 mM EDTA Na2.2H2O, 86 mM NaCl, 10 mM metabisulfite, 1%PVP-10, pH adjusted to 7.5). *Vicia faba* was used as the internal reference standard (2C = 26.90 pg DNA). The nuclear suspension obtained was filtered with a nylon filter and 50 µg/ml of propidium iodide was added to stain the DNA. 50 µg/ml of RNAse was added to avoid staining of double stranded RNA. All samples were analyzed in a Partec CyFlow Space flow cytometer and the results were checked using Partec FloMax software v2.4d (Partec GmbH, Münster, Germany). At least 5000 particles were analysed per sample. For each sample, the DNA index was calculated and the nuclear DNA content of *J. thurifera* was estimated by multiplying the DNA index by the known genome size of the *V. faba*, using the formula 1 pg = 978 Mbp.

### Microsatellites amplification

Due to the larger number of alleles per locus, the discriminative power of a locus is potentially higher for polyploids than for diploids and the successful fingerprinting of polyploids requires substantially fewer loci [17]. An initial set of putative polymorphic 11 nuclear microsatellite (nSSR) loci developed for *J. thurifera* by Genoscreen (Lille, France) was tested (Table 1). Six out of these 11 primer pairs amplified successfully the targeted loci and showed polymorphism. These six polymorphic primers pairs were therefore used in this study (Table 1). These primers were developed during this study and have not been tested for cross-amplification with other species.

**Table 1:** Characteristics of the 11 primers tested for *J. thurifera* genotyping: locus name, forward and reverse primer sequences, motif, size, annealing temperature and fluorescent dye used.

Locus name	Forward primer sequence	Reverse primer sequence	Motif	Allele size range (bp)	Annealing T (°C)
JT_01	AATCCATCACATGCCATCTTT	CCCTCATAAGAATCAATGAGATCC	TA_6_	120–182	57
JT_04	CCAAGGAATGATCTAACCTTTGAA	TGGGATGCATATCTTATCTTCCT	AGA_7_	174–219	55
JT_30	AATCCCCTATCCTTGCCAGT	TCAACAATATCAGCAAGTAATGAGA	TCT_10_	128–220	57
JT_33	GAGCTTCCTTTGTAGATTTTGGG	GTAAGAAGACACCACTCAGTCGAT	CT_11_	177–285	57
JT_40	GGCCGCATGATCCATTACT	TCGTAACGTAATGACATGTATAGTGC	CA_20_	98–150	54
JT_46	TGAGATCACCTACTTCCTAGTGGA	CCACCAAGGGCATAGAGTTC	AGG_7_	174–237	54
JT_02	TTCTTCCTCACTTTTGTTGCC	AGGTGCTAGGGGTGCTTGTA	GAA_8_	148	55
JT_03	ACCCTCTATAAGGATGCTACCATGA	AAAAGATAGATTGATAAGTTGAAAGGG	CTT_8_	140	55
JT_34	CATGCATGGGTTATAATAATAGAGATA	TGGGCACAAATTTTAGTGTAATG	AG_12_	110	55
JT_37	GATGTTTGTATCATATCCTTGATTGG	TCCACACCTATCGGGTTCAT	GT_14_	123	55
JT_38	CCAACAAGCCTCCACCCTAT	CAAGTTTGGAAAGTGTGGTCA	AC_14_	114	55

Above: loci used in genotyping, below: loci that did not show polymorphism.

Multiplex reactions were prepared using primers with similar annealing temperature that amplify fragments of different size. Polymerase chain reaction (PCR) was performed in a total volume of 20 µl, containing 20 ng of DNA template, 2× QIAGEN Multiplex PCR Master Mix and 1 µM of each forward (F) and reverse (R) primers and RNase-free water. All PCR were run in a Gene Amp 9700 (Applied Biosystems) using the following program: Initial denaturation step for 15 min at 95°C, followed by 40 cycles of 30 s at 94°C, 90 s at locus-specific annealing temperature (see Table 1) and 60 s at 72°C and a final extension step for 30 min at 60°C and cooling down to 4°C. PCR products were checked on 2% TBE agarose gels stained with RedSafe. PCRs that did not produce bands or that had different size were repeated. The amplification products were combined with formamide and a size standard (GeneScan-500 LIZ, Applied Biosystems) and separated on a 3730 ABI automated sequencer. Sample profiles were scored manually using GeneMarker v2.4 (Applied Biosystems).

### Data analysis

The quantification of genetic diversity in polyploids can be problematic because of the difficulties in assigning the correct allele dosage for each loci and individual [18]–[20]. Although it has been proposed that allele dosage could be estimated in tetraploids on the basis of electropherogram peak height [19], this was not possible for the studied species. Fixed heterozygosity is common in polyploidy species and homozygote individuals are rare due to the presence of at least two allele variants associated with different isoloci [19]. Due to these deviations from diploid meiotic behaviour, indices such as expected heterozygosity (H_e_), cannot be used to study genetic diversity in polyploids such as *J. thurifera* [19], [21], [20]. Also, the common karyotype homogeneity of gymnosperms impedes to know if *J. thurifera* is an autopolyploid or allopolyploid species. The effective number of alleles per locus (Ae′) and heterozygosity within each population (Hs) were calculated using GenoDive, a software that allows analysing polyploids with unknown dosage of alleles [22]. For every incomplete marker phenotype (e.g. AB) it is possible to calculate the likelihood of observing that phenotype given some set of allele frequencies, by combining the likelihoods of the underlying genotypes (AAAB, AABB, or ABBB). This correction uses a maximum likelihood method based on an algorithm that calculates for each locus and population the likelihood of different allele frequencies for all observed phenotypes until convergence is reached.

The multilocus data was transformed to a binary matrix of presence/absence of each allele for each individual [18], [19], [23], which was used for further analysis with GenAlex 6.5 [24]. Total number of alleles, genetic diversity and Shannon's information index for each population, and the number of private alleles per population and region were calculated using this approach. The genetic differentiation between populations was determined using phiPT, a measure that allows intra-individual variation to be suppressed and is therefore ideal for comparing codominant and binary data, with 10,000 permutations [25]. Analysis of molecular variance (AMOVA) among and within populations and geographical regions was performed using GenAlex [24], [26]. A Mantel test, as implemented in GenAlex, was performed using genetic and geographical distances to test for isolation by distance [27]. Fine-scale genetic structure was investigated using spatial autocorrelation analysis in GenAlEx 6.5 [24]. The spatial autocorrelation coefficient (*r*) was computed using the geographic distance and the multilocus genetic distance between individuals. Tests for statistical significance were conducted by random shuffling (999 times) of individual geographic locations to define the upper and lower bounds of the 95% confidence interval for each distance class, and estimating 95% confidence intervals around mean *r* values by bootstrapping pair-wise comparisons within each distance class (1000 repeats). Analyses were performed using the variable distance classes option based on the geographical distances estimated in each population.

The original genotypic data matrix was used to perform a PCA using the Bruvo distance to explore the genetic similarity between samples. This analysis was performed with PolySat [28], an R package for polyploid microsatellite analysis in ecological genetics.

The genetic structure of each population was inferred with the BAPS software [29] following a model-based Bayesian assignment that allows mixed ancestry of individuals (admixture model).

## Results

### Ploidy level in Juniperus thurifera

Flow cytometry from individuals of the two *Juniperus* species resulted in clear fluorescence peaks. *Juniperus phoenicea*, a known diploid species, had 2C of ≈20 pg, while *J. thurifera* had 2C of ≈40 pg, revealing that the genomic size of *J. thurifera* is double than that in *J. phoenicea*. Knowing that *J. phoenicea* is a diploid species, the value observed for *J. thurifera* would correspond to a tetraploid species. The 2C values obtained were the same for all *J. thurifera* samples, meaning that ploidy levels did not experience variation among regions or populations. The values obtained were similar to those published by Romo et al. [30] for *J. thurifera* and correspond to a tetraploid species.

### Genetic diversity

A total of 153 alleles were amplified from the 6 microsatellite loci. All 6 loci were found to be highly polymorphic and displayed up to 4 alleles per sample, in agreement with the ploidy level detected by flow cytometry. The number of different alleles found within each population (An) was generally higher in the populations from Spain than in Morocco, while the average number of effective alleles per locus was similar in all populations (Table 2). No significant differences between regions were found for any of these diversity estimators (Wilcoxon test, *P* = 0.12). All populations showed also similar high values of heterozygosity (Table 2). Private alleles were found in all populations from Spain and in two populations from Morocco (Tizi Techt and Azzaden Oussem). The number of private alleles per population found in each region was significantly higher in the Iberian Peninsula (Wilcoxon test, *P* = 0.01). Significantly higher values were also found in the Iberian Peninsula for the Shannon's information index (I) and the estimator of genetic diversity (H) (Table 2, Wilcoxon tests, *P* = 0.05 and *P* = 0.04 respectively).

**Table 2:** Diversity of the studied populations of *Juniperus thurifera* obtained from the analysis of 6 nuclear microsatellite loci.

Country	Population	N	An	Ae′	PA	Hs	I	*H*
Morocco	Oukaimeden 1	19	88	6.272	0	0.802	0.213	0.129
	Tizi Techt	22	80	5.230	1	0.793	0.190	0.115
	Oukameiden 2	20	71	5.580	0	0.792	0.181	0.112
	Matat	19	73	5.651	0	0.800	0.189	0.117
	Azzaden Oussem	31	82	5.019	1	0.773	0.187	0.113
Spain	LunaA	21	74	6.075	3	0.811	0.193	0.120
	LunaB	30	101	6.672	4	0.822	0.225	0.135
	Abejar	24	88	6.146	1	0.795	0.211	0.129
	Cabrejas	24	100	6.693	3	0.802	0.222	0.134
	Lanaja	27	90	5.753	1	0.789	0.206	0.124
	Monegros	24	80	5.419	4	0.776	0.197	0.120

N =  number of individuals, An =  number of alleles in each population, Ae′ =  average number of effective alleles per locus, PA =  number of private alleles per population, Hs =  heterozygosity within populations, I =  Shannon's Information Index, *H* =  genetic diversity.

From the 153 alleles observed, there were 105 alleles (69%) shared by all populations. The remaining alleles were exclusively found in populations of Spain or in populations of Morocco. The number of private alleles found in Spain was higher than in Morocco (Table S2). 40 private alleles (26%) were found only in populations from Spain while only 8 private alleles (5%) were found exclusively in the populations from Morocco. From the six loci used in this survey, JT33 was the locus with the highest number of private alleles, with 12 private alleles in Spain and 4 in Morocco. The locus JT04 was the only one that did not display private alleles in Spain. In Morocco, the loci JT01 and JT40 did not display private alleles and the loci JT04 and JT30 just had one private allele (Table S2).

### Population differentiation and genetic structure

Pairwise PhiPT genetic distances ranged between 0.004 (Azzaden Oussem/Matat) and 0.127 (Azzaden Oussem/Luna B). The PhiPT distances between Azzaden Oussem and Matat (0.004) and between Tizi Techt and Matat (0.012) were not statistically significant (Table 3). The Analysis of Molecular Variance (AMOVA) based on PhiPT values indicated that most of the genetic diversity occurred within populations (90%) while the variability among populations and among regions contributed 4% and 6%, respectively, to the observed genetic diversity (Table 4).

**Table 3:** Pairwise PhiPT genetic distances between the studied populations.

	Oukameiden 1	Tizi Techt	Oukameiden 2	Matat	Azzaden Oussem	LunaA	LunaB	Abejar	Cabrejas	Lanaja	Monegros
Oukameiden 1		0.001	0.004	0.001	0.001	0.001	0.001	0.001	0.001	0.001	0.001
Tizi Techt	0.070		0.001	0.118	0.007	0.001	0.001	0.001	0.001	0.001	0.001
Oukameiden 2	0.030	0.043		0.003	0.003	0.001	0.001	0.001	0.001	0.001	0.001
Matat	0.066	0.012	0.051		0.282	0.001	0.001	0.001	0.001	0.001	0.001
Azzaden Oussem	0.042	0.022	0.026	0.004		0.001	0.001	0.001	0.001	0.001	0.001
LunaA	0.084	0.115	0.099	0.091	0.097		0.001	0.001	0.001	0.001	0.001
LunaB	0.105	0.125	0.122	0.112	0.127	0.032		0.001	0.001	0.001	0.001
Abejar	0.073	0.094	0.088	0.077	0.085	0.046	0.056		0.008	0.004	0.002
Cabrejas	0.087	0.084	0.096	0.075	0.098	0.061	0.042	0.022		0.003	0.002
Lanaja	0.068	0.090	0.106	0.074	0.086	0.055	0.058	0.023	0.026		0.012
Monegros	0.084	0.076	0.106	0.068	0.093	0.065	0.048	0.034	0.029	0.019	

PhiPT values are shown below diagonal. Probability based on 1000 permutations is shown above diagonal.

**Table 4:** Analysis of molecular variance (AMOVA) showing the partitioning of genetic variation within and between regions of *J. thurifera*.

Source of variation	df	SS	MS	Est. Var.	%	*P*
Among Regions	1	99.898	99.898	0.631	5.80	<0.001
Among Population	9	172.093	19.121	0.394	3.60	<0.001
Within Population	250	2451.678	9.806	9.806	90.50	<0.001

(df =  degree of freedom, SS =  sum of squares, MS mean squares, Est. var. =  estimate of variance, % =  percentage of total variation, P-value is based on 1000 permutations).

**Figure 1:**
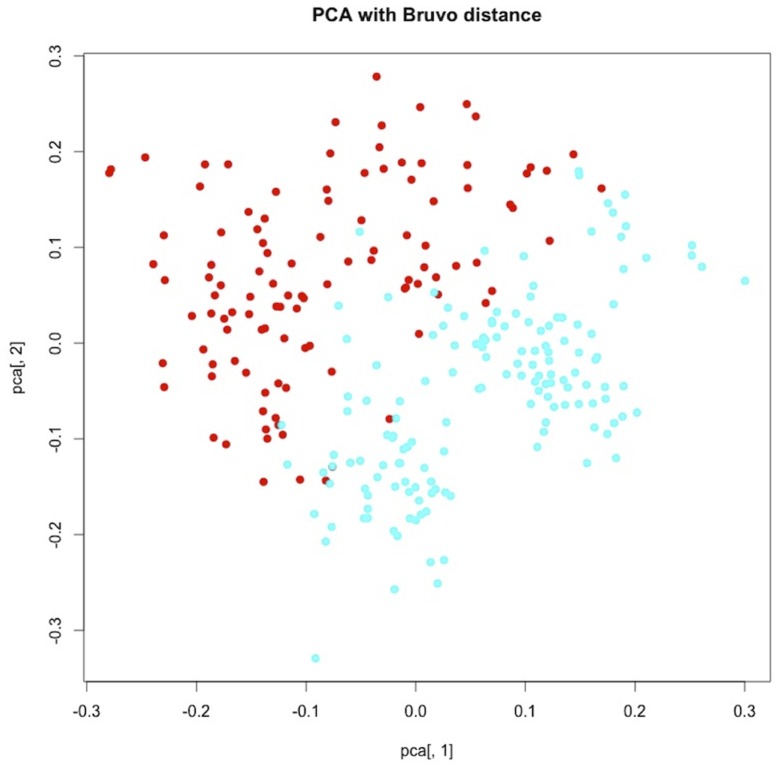
Principal Component Analysis (PCA) of the 261 individuals based on genotyping with 6-microsatellite loci. Red: samples from Morocco, blue: samples from Spain.

The Principal Component Analysis (PCA) separated samples from Spain and Morocco into two groups along the first axis, although there was some overlap between them (Figure 1). The Mantel test based on Euclidean distances among populations revealed a weak but significant correlation among genetic and geographic distances (R^2^ = 0.048, *P* = 0.01). Spatial autocorrelation was detected only for the smallest distance class (Figure S1).

**Figure 2:**
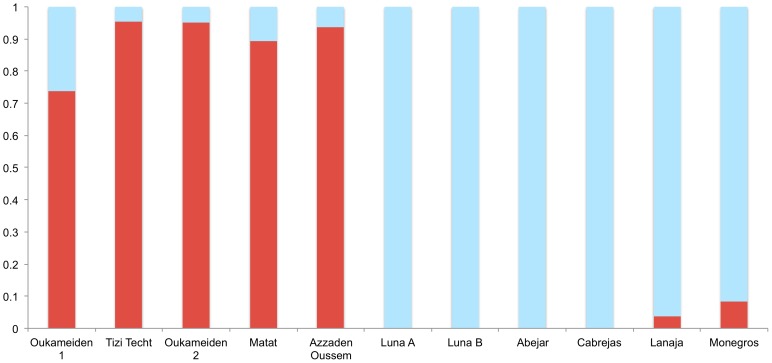
Means of assignment coefficients to each group (K = 2) per population according to BAPS. Populations are ordered by their origin. Morocco: Oukameiden 1, Tizi Techt, Oukameiden 2, Matat, Azzaden Oussem. Spain: Luna A, Luna B, Abejar, Cabrejas, Lanaja, Monegros.

Bayesian analysis using BAPS showed the existence of 2 ancestral groups (K = 2) that were dominant in each of the two regions, Morocco and Spain (Figure 2). The two clusters were easily distinguished although there were a few migrants in both groups (2% of individuals in Morocco and 9% of individuals in Spain). Genetic admixture between the two ancestors was not detected.

## Discussion

The analysis of 11 populations of *J. thurifera* using 6 polymorphic nSSR revealed high values of genetic diversity within populations and a clear separation between the populations of Morocco and Spain. This is the first study of the polyploid *J. thurifera* using nuclear microsatellites. Six primer pairs were developed that are useful for genotyping both *J. thurifera* subspecies. Our results show some geographical differentiation following the current division of this species into two different subspecies separated by the Strait of Gibraltar: *J. thurifera* subsp. *africana* in Africa and *J. thurifera* subsp. *thurifera* in Europe [9], [10], [7], [4], although some genetic flow was detected between both regions.

Polyploidy is a rare event in gymnosperm species. The genus *Juniperus* is the gymnosperm genus with most number of cases of natural polyploidy. There are three known cases of natural tetraploidy (*J. chinensis*, *J. sabina* and *J. thurifera*) and others cases of non-natural triploidy and tetraploidy (*J. chinensis*, *J. sabina, J. squamata, J. virginiana*). But *J. thurifera* is the only species exclusively polyploidy in the genus [30]. All the analysed *J. thurifera* individuals had the same genome size and no differences in genome size were found between *J. thurifera* subsp. *thurifera* and *J. thurifera* subsp. *africana*. These results are in accordance with the survey published by Romo et al. [30] and suggest that polyploidy in *J. thurifera* happened early in its evolutionary history.

The high values of within population genetic diversity found in *J. thurifera* are in accordance with other studies in conifers [31], [32], [10], [4], [33]. Wind-pollination and dioecy might contribute largely to this high level of genetic diversity within populations. The two studied regions accounted for 6% of the variability, while between populations variation only accounted for 4% of the total genetic diversity. Weak spatial genetic structure of individuals was revealed by spatial autocorrelation analysis, likely due to the high within population genetic diversity observed for this species. The PhiPT values also showed a weak genetic differentiation between populations within each of the studied regions as previously shown by Terrab et al. [4] using AFLPs. This lack of genetic differentiation between populations within regions might be explained by active seed dispersal by animals. Thrushes (*Turdus* spp.), rabbits (*Oryctolagus cuniculus*), red foxes (*Vulpes vulpes*), stone martens (*Martes foina*) and even domestic sheeps (*Ovis aries*) consume *J. thurifera* fleshy fruits and can be effective seed dispersers for this species at medium and long distances [34]. A larger distribution of *J. thurifera* during cold periods of the Pleistocene that allowed gene flow between the currently fragmented populations might also have contributed to the low genetic structure detected within regions [35], [4].

We did find evidences of genetic differentiation between the Moroccan (subsp. *africana*) and Spanish (subsp. *thurifera*) populations of *J. thurifera*, likely due to the geographical barrier imposed by the Strait of Gibraltar [4], [36]. However, this is not an impervious barrier and the PCA indicates not only isolation by distance but also a possible gene flow between populations of both sides of the Strait of Gibraltar. This might happen through seeds transported by migrant thrushes, which are the main seed disperser of *J. thurifera* [37], [34].

*J. thurifera* populations were more diverse in Spain than in Morocco as shown by the significantly higher genetic diversity and the higher number of private alleles found in this region. The higher number of private alleles found in Spain might reflect the existence of glacial refugia, as it has been shown for other conifers [38], [39] or a higher historical, or contemporary, gene flow in Europe [4]. Also, the lower genetic diversity in Morocco might be explained by the colonization history of *J. thurifera*, which is a species that originated in Europe and spread to North Africa from the Iberian Peninsula [10]. Our results indicate a possible loss of genetic diversity during migration due to a bottleneck effect, environmental filters in North Africa or genetic drift. Posterior isolated evolution of the migrant genotypes would lead to the current genetic differentiation between Morocco and Spain.

To sum up, genotyping of *J. thurifera* using nuclear microsatellites supports the separation of Moroccan and Spanish populations into two differentiated genetic groups that correspond to the proposed subspecies *africana* and *thurifera*. A high number of private alleles were found in Spain, which might indicate that these populations were part of genetic refugia during glaciations or reflect the longest isolation of the Moroccan populations. Within population genetic diversity was high in both regions while population structure was weak, which might reveal an active transport of seeds between populations and also across the Strait of Gibraltar.

## Supporting Information

Figure S1 **Correlogram from spatial autocorrelation analysis using the correlation coefficient** ***r*** **by Smouse & Peakall (1999), and variable distance classes.** 95% confidence error bars for r were estimated by bootstrapping over pairs of samples; dashed lines (U, L) represent upper and lower bounds of a 95% CI for r generated under the null hypothesis of random geographic distribution.
(DOCX)

Table S1 **Geographic locations of the 11 populations of** ***J. thurifera*** **in study.**
(DOCX)

Table S2 **Number of alleles per locus: total number of alleles, alleles shared between both regions and private alleles found for each region.**
(DOCX)
