# Correction: Genetic Diversity and Differentiation of *Juniperus thurifera* in Spain and Morocco as Determined by SSR

**DOI:** 10.1371/journal.pone.0126042

**Published:** 2015-05-12

**Authors:** Helena Teixeira, Susana Rodríguez-Echeverría, Cristina Nabais

In the Funding section, an additional grant number from the funder Fundação para a Ciência e Tecnologiais is missing. The additional grant number is: PEst-OE/BIA/UI4004/2014.

## References

[1] Teixeira H, Rodríguez-Echeverría S, Nabais C (2014) Genetic Diversity and Differentiation of *Juniperus thurifera* in Spain and Morocco as Determined by SSR. PLoS ONE 9(2): e88996 doi:10.1371/journal.pone.0088996 24533164 10.1371/journal.pone.0088996 PMC3923062

