# Supplementary material for: Genetic Diversity and Differentiation of Juniperus thurifera in Spain and Morocco as Determined by SSR
Source: PLoS One. 2014 Feb 12;9(2):e88996. doi: 10.1371/journal.pone.0088996 (PMC3923062; doi:10.1371/journal.pone.0088996)
Supplement: Figure S1 — Correlogram from spatial autocorrelation analysis using the correlation coefficient r by Smouse & Peakall (1999), and variable distance classes. 95% confidence error bars for r were estimated by bootstrapping over pairs of samples; dashed lines (U, L) represent upper and lower bounds of a 95% CI for r generated under the null hypothesis of random geographic distribution. (DOCX) [file pone.0088996.s001.docx]

**Figure S1**

Reference

Smouse PE, Peakall R (1999) Spatial autocorrelation analysis of individual multiallele and multilocus genetic structure. Heredity 82: 561–573.
