# Supplementary material for: Genetic Diversity and Differentiation of Juniperus thurifera in Spain and Morocco as Determined by SSR
Source: PLoS One. 2014 Feb 12;9(2):e88996. doi: 10.1371/journal.pone.0088996 (PMC3923062; doi:10.1371/journal.pone.0088996)
Supplement: Table S1 — Geographic locations of the 11 populations of J. thurifera in study. (DOCX) [file pone.0088996.s002.docx]

Table S1.

| Country | Populations | N | Coordinates | Altitude (m) |
| --- | --- | --- | --- | --- |
| Morocco | Oukaimeden 1 | 19 | 31.19997 N/ 7.88237 W | 2600 |
|  | Tizi Techt | 22 | 31.17007 N/ 7.97286 W | 2033 |
|  | Oukameiden 2 | 20 | 31.20028 N/ 7.87570 W | 2540 |
|  | Matat | 19 | 31.15095 N/ 7.95622 W | 2060 |
|  | Azzaden Oussem | 31 | 31.11579 N/ 7.95656 W | 2331 |
| Spain | Luna A | 21 | 42.87560 N/ 5.83618 W | 1350 |
|  | Luna B | 30 | 42.87814 N/ 5.85954 W | 1239 |
|  | Abejar | 24 | 41.78647 N/ 2.77123 W | 1100 |
|  | Cabrejas | 24 | 41.78947 N/ 2.77254 W | 1200 |
|  | Monegros | 27 | 41.81144 N/ 0.54535 W | 1000 |
|  | Lanaja | 24 | 41.66963 N/ 0.35169 W | 1365 |
