# Supplementary material for: Genetic Diversity and Differentiation of Juniperus thurifera in Spain and Morocco as Determined by SSR
Source: PLoS One. 2014 Feb 12;9(2):e88996. doi: 10.1371/journal.pone.0088996 (PMC3923062; doi:10.1371/journal.pone.0088996)
Supplement: Table S2 — Number of alleles per locus: total number of alleles, alleles shared between both regions and private alleles found for each region. (DOCX) [file pone.0088996.s003.docx]

**Table S2**

|  | Shared alleles | Private alleles | | Total |
| --- | --- | --- | --- | --- |
| Locus |  | Spain | Morocco |  |
| JT01 | 22 | 7 | 0 | 29 |
| JT04 | 8 | 0 | 1 | 9 |
| JT30 | 23 | 6 | 1 | 30 |
| JT33 | 24 | 12 | 4 | 40 |
| JT40 | 13 | 12 | 0 | 25 |
| JT46 | 15 | 3 | 2 | 20 |
| Total | 105 | 40 | 8 | 153 |
